# Syntheses of Pyrene-4,5-dione and Pyrene-4,5,9,10-tetraone

**DOI:** 10.1021/acs.joc.5c01542

**Published:** 2025-10-04

**Authors:** Omolola Balogun, Besan Khader, Tetyana Ignatova, Aleksandrs Prokofjevs

**Affiliations:** † Department of Chemistry, North Carolina A&T State University, Greensboro, North Carolina 27411, United States; ‡ Nanoscience Department, The Joint School of Nanoscience & Nanoengineering, 14616 University of North Carolina, Greensboro, North Carolina 27401, United States

## Abstract

Improved gram scale synthesis procedures for the preparation of pyrene-4,5-dione and pyrene-4,5,9,10-tetraone are reported. Pyrene-4,5-dione has been synthesized using potassium persulfate as the oxidant and RuO_2_·*n*H_2_O as the catalyst in the biphasic CH_2_Cl_2_/H_2_O solvent mixture containing K_2_CO_3_ as the base. We also developed several procedures for multigram scale oxidation of pyrene-4,5-dione to pyrene-4,5,9,10-tetraone, eliminating the need for chromatographic purification of the poorly soluble tetraone product.

## Graphical abstract

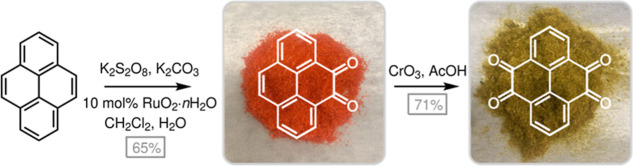

First reported in 1937,^1^ pyrene-4,5-dione (**1**) and pyrene-4,5,9,10-tetraone (**2**) have since become valuable building blocks for construction of extended polyaromatic systems,^2^ specialty polymers,^3^ ligands,^4^ and components of energy storage devices.^5^ Most substitution reactions of the pyrene core occur at positions 1, 3, 6, and 8,^6^ with a few significant exceptions targeting positions 2 and 7 with high selectivity.^7^ Reactions involving positions 4, 5, 9, and 10 of the unsubstituted pyrene are much less common, making dione **1** and tetraone **2** the most significant entrance points for the synthesis of pyrene derivatives substituted in the K-region.

To this end, ruthenium-catalyzed oxidation of pyrene remains the chief method for synthesizing K-area quinones. The initial report on RuO_4_/periodate oxidation of pyrene to 4,5-dione **1** ^8^ remained relatively unnoticed until 2005, when it was transformed into a more practical preparative procedure by Harris et al., employing RuCl_3_·*n*H_2_O/NaIO_4_ in a CH_2_Cl_2_/CH_3_CN/H_2_O solvent mixture and also enabling one-pot synthesis of tetraone **2**.^9^ The highly desirable single-step procedure presented inherent limitations, including poor scalability and cumbersome chromatographic isolation of the target products, particularly problematic for the poorly soluble tetraone **2**. Subsequent studies aimed at improving the original procedure by Harris, with Bodwell^10^ reporting the first truly scalable protocol using an *N*-methylimidazole additive to facilitate the workup. To the best of our knowledge, no substantial improvements to the original synthesis of pyrene-4,5,9,10-tetraone have been reported aside from the multistep indirect route.^11^

High oxidation state ruthenium oxidations are widespread in organic synthesis with RuO_4_ offering unmatched reactivity in aromatic ring oxidative transformations. While most RuO_4_-catalyzed reaction protocols utilize NaIO_4_ as the stoichiometric oxidant, other oxidants have also been used occasionally.^12^ Seeking economical access to multigram quantities of the pyrene K-region quinones, we investigated the possibility of using potassium persulfate K_2_S_2_O_8_ as the replacement for the expensive periodate in the Ru-catalyzed oxidation of pyrene to the 4,5-dione. In our early experiments, we confirmed that pyrene reacts with persulfates under acidic conditions (i.e., in AcOH) in the absence of ruthenium to produce a mixture of the undesired pyrene-1,6- and pyrene-1,8-diones along with their subsequent degradation products. Pyrene was found to be resistant to noncatalyzed oxidation by the persulfate under neutral or basic conditions, enabling the pathway for the Ru-catalyzed oxidation with minimal interference.

To our delight, attempts to oxidize pyrene with K_2_S_2_O_8_/RuCl_3_·*n*H_2_O in the H_2_O/CH_2_Cl_2_/MeCN mixture resulted in formation of the desired pyrene-4,5-dione, although achieving satisfactory conversion and reproducibility proved challenging under the initial conditions. Subsequent experiments showed that addition of a nonoxidizable base to the reaction mixture was essential for increasing the reaction efficiency. While NaHCO_3_ was used in our initial studies, K_2_CO_3_ is employed in the optimized procedure due to its higher solubility in water, greatly improving the stirring in large scale preparations.

RuO_4_-mediated reactions, including previously reported pyrene oxidations, are almost exclusively run in the ternary H_2_O/CH_2_Cl_2_/MeCN mixture, reflecting the need to simultaneously dissolve the inorganic oxidant (most commonly NaIO_4_) and the organic substrate, as well as ensure RuO_4_ transfer between the phases. It has also been suggested that MeCN plays a stabilizing role for ruthenium species, improving catalytic efficiency.^13^ We were thus quite surprised to observe that eliminating MeCN cosolvent had a major positive effect on formation of pyrene-4,5-dione, while also simplifying product isolation. While other solvents such as THF and EtOAc have also been tested, the binary CH_2_Cl_2_/H_2_O mixture showed excellent performance, while also making extraction of the pyrene-4,5-dione product at the end of the reaction nearly effortless.

The third key improvement to the oxidation protocol was using Ru­(IV) oxide hydrate RuO_2_·*n*H_2_O as the RuO_4_ precursor instead of the more commonly used RuCl_3_·*n*H_2_O. While RuCl_3_·*n*H_2_O often showed satisfactory performance, a few batches of the chloride acquired from commercial sources led to an unusually slow reaction in a reproducible manner. In contrast, ruthenium­(IV) oxide hydrate RuO_2_·*n*H_2_O showed excellent performance irrespective of the source, while also being easier to handle as compared to the deliquescent chloride.

The above improvements allowed formulation of a straightforward experimental procedure that has since been repeated dozens of times by multiple researchers in our laboratory, most commonly starting with 10 g of pyrene (Scheme 1). The optimized protocol involves heating a mixture of pyrene, K_2_S_2_O_8_, K_2_CO_3_, and RuO_2_·*n*H_2_O in a biphasic CH_2_Cl_2_/H_2_O solvent under reflux for 14–24 h, followed by simple extraction to separate dione **1** from the inorganic byproducts. Another distinguishing feature of our protocol is the very high purity of the dione product, eliminating the need for chromatographic purification for most applications. In fact, the only pyrene-derived byproduct we were able to detect is 2,2′,6,6′-biphenyltetracarboxylic acid, reliably trapped in the highly basic aqueous phase during the extraction process. No trace of pyrene-4,5,9,10-tetraone (**2**) was detected despite considerable experimentation, and the yield of dione **1** does not noticeably decrease with extended reaction times, suggesting formation of the 2,2′,6,6′-biphenyltetracarboxylic acid byproduct early in the process.

**Scheme 1:**
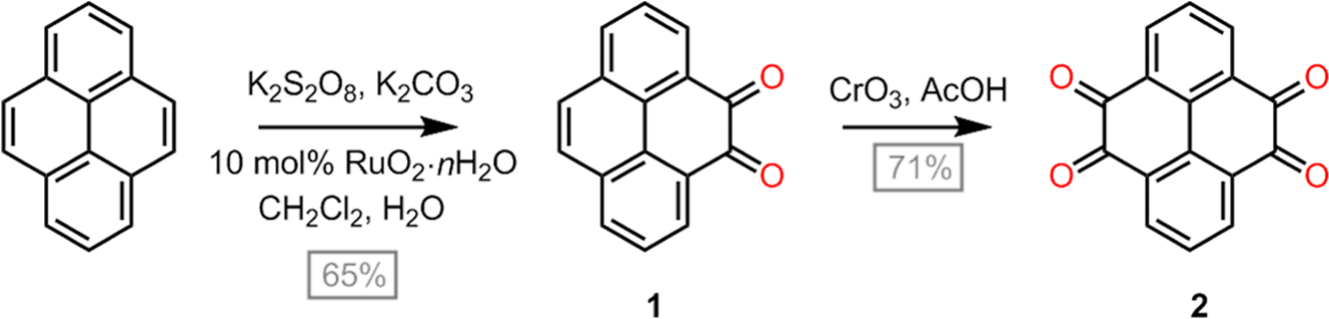
Syntheses of Pyrene-4,5-dione and Pyrene-4,5,9,10-tetraone

Having developed a reliable procedure for the synthesis of the dione, we decided to utilize this compound as the entry point for the synthesis of tetraone **2**. Despite the added step, using dione **1** as the starting point in the synthesis of **2** has a number of advantages. First, while directing the initial reactivity in the K-region of unsubstituted pyrene is nontrivial, necessitating the use of RuO_4_ or related reagents, dione **1** is substantially activated toward subsequent oxidation at the 9,10-position compared to the starting pyrene. This stems from the fact that the aromatic system of pyrene-4,5-dione (**1**) is more similar to that of phenanthrene rather than of the starting pyrene. Consequently, oxidation of dione **1** to tetraone **2** does not need to rely on Ru catalysis at all. Second, conditions required for the initial pyrene oxidation to dione **1** are not well-suited for subsequent oxidation to tetraone **2**, and attempts to combine both oxidation steps into a single pyrene-to-tetraone protocol are more likely to lead to byproduct formation. Poor solubility of both tetraone **2** and the byproducts makes subsequent chromatographic purification tedious, as anyone familiar with the previously reported tetraone preparation protocols will attest to.

Consequently, we formulated three separate experimental protocols providing access to practical quantities of tetraone **2** in high purity starting with dione **1** and a choice of the oxidant: NaIO_4_, H_5_IO_6_, or CrO_3_. In all three experimental protocols, the pure tetraone **2** product is isolated from the reaction mixture in high purity by merely diluting the reaction mixture with water. While we could not effect further oxidation of 4,5-dione **1** using RuO_2_·*n*H_2_O/K_2_S_2_O_8_ mixtures under a variety of conditions, a RuO_2_·*n*H_2_O/NaIO_4_ mixture in MeCN/H_2_O delivered the tetraone in 62% yield after merely 2 h at room temperature. While the conventional mechanistic picture suggests that “RuO_4_” is the key oxidizing agent, the stark contrast between the outcomes of the RuO_2_·*n*H_2_O/K_2_S_2_O_8_ and RuO_2_·*n*H_2_O/NaIO_4_ protocols suggests that tetraone formation cannot be explained by the action of RuO_4_ alone. Seeking a ruthenium-free version of the oxidation protocol, we observed that H_5_IO_6_ in AcOH is also quite efficient in the oxidation of dione **1**, furnishing tetraone **2** in 57% yield. While H_5_IO_6_ was previously applied to K-region oxidations in 2,7-di-*tert*-butylpyrene and its derivatives,^14,15^ its use for oxidation of **1** to **2** has not been reported in the literature. Our most favored tetraone synthesis protocol employs refluxing dione **1** with anhydrous CrO_3_ in glacial acetic acid, delivering highly pure **2** in 71% yield upon diluting the reaction mixture with water.

In summary, we are reporting a set of highly convenient protocols for preparation of pyrene-4,5-dione **1** and pyrene-4,5,9,10-tetraone **2** on a multigram scale.

## Experimental Section

### Pyrene-4,5-dione

A 1 L round-bottom flask equipped with a highly efficient stirring magnet and a reflux condenser was charged with solid pyrene (10.0 g, 49.6 mmol), K_2_S_2_O_8_ (95.0 g, 0.35 mol), K_2_CO_3_ (95.0 g, 0.48 mol), and RuO_2_·*n*H_2_O (1.00 g, 7.51 mmol). Water (300 mL) and CH_2_Cl_2_ (300 mL) were added, and the resulting dark brown slurry was stirred at mild reflux (the oil bath surrounding the reaction flask was kept at 48 °C) for 14–24 h. For most commercial persulfate batches tested, the reaction was complete in 14 h, as evidenced by the complete disappearance of pyrene by TLC (10:1 CH_2_Cl_2_:hexanes, *R*_f_ = 0.63) and ^1^H NMR analysis. We have occasionally encountered commercial K_2_S_2_O_8_ batches composed almost entirely of very large (>3 mm) crystals. In those cases, it is advisable to grind the persulfate to a fine powder first, increase the K_2_S_2_O_8_ loading to 110 g, and extend the reaction time to 24 h.

The reaction mixture was subsequently treated with 50 mL of 2 M aqueous Na_2_SO_3_ to quench the residual Ru­(VIII) (optional precautionary step), the brightly colored organic layer was separated, and the aqueous layer was extracted with additional CH_2_Cl_2_ (3 × 120 mL). The combined CH_2_Cl_2_ extracts were dried with anhydrous MgSO_4_, filtered, and concentrated under reduced pressure to afford a bright orange solid (7.58 g, 65%). For most commercial persulfate batches tested, the product after concentration does not contain any observable traces of pyrene or other impurities as evidenced by ^1^H NMR or TLC, and it is sufficiently pure for most applications without additional purification. Occasionally, when a lower quality persulfate starting material was used, a slight trace of unreacted pyrene can be detected in the crude product, in which case the material can be recrystallized from refluxing glacial AcOH (85 mL per 1 g of the crude dione) to afford very high-purity pyrene-4,5-dione in the form of long orange needles. The recrystallized product can be conveniently dried in a conventional heating oven kept at 120 °C to remove the residual AcOH.

^1^H NMR (400 MHz, CDCl_3_): δ 8.46 (dd, *J* = 7.5, 1.3 Hz, 2H), 8.15 (dd, *J* = 8.0, 1.3 Hz, 2H), 7.82 (s, 2H), 7.74 (dd, *J* = 8.0, 7.5 Hz, 2H); ^1^H NMR (400 MHz, DMSO-*d*_6_): δ 8.36–8.30 (m, 4H), 8.02 (s, 2H), 7.84 (dd, *J* = 7.9, 7.4 Hz, 2H); ^13^C­{^1^H} NMR (101 MHz, CDCl_3_): δ 180.4, 135.8, 132.0, 130.2, 130.1, 128.4, 128.0, 127.3; ^13^C­{^1^H} NMR (101 MHz, DMSO-*d*_6_): δ 179.2, 134.9, 131.6, 130.6, 128.6, 127.9, 127.8, 127.1; HRMS (ESI/Q-TOF) calcd for C_16_H_9_O_2_^+^ (M + H)^+^, 233.0597; found, 233.0596.

### Pyrene-4,5,9,10-tetraone from Pyrene-4,5-dione Using CrO_3_ (Recommended Procedure)

A 250 mL round-bottom flask fitted with a stirring magnet and a reflux condenser was charged with pyrene-4,5-dione (5.0 g, 21.6 mmol) and 150 mL of glacial acetic acid. The resulting orange slurry was stirred at reflux (the oil bath surrounding the reaction flask was kept at 130 °C) for 10 min. Solid CrO_3_ (12.0 g, 0.12 mol) was added in portions over the course of 3 min, after which the reaction mixture was refluxed overnight. The dark green solution was poured into 300 mL of H_2_O, and the crude product was collected by filtration and dissolved in 15 mL of 96% H_2_SO_4_. Pouring the sulfuric acid solution into 300 mL of H_2_O precipitated the tetraone, which was collected by filtration and washed on the filter with copious amounts of H_2_O until neutral pH. The wet product was dried in the heating oven at 120 °C to afford 4.0 g (71%) of a custard-yellow solid, pure by TLC and ^1^H NMR. The tetraone product can be further recrystallized from refluxing benzonitrile (40 mL per 1 g of the tetraone) to produce long needles, which are then dried in the oven at 120 °C to remove the residual crystallization solvent. No melting point could be measured due to facile sublimation of the product at high temperatures.

^1^H NMR (400 MHz, CDCl_3_): δ 8.52 (d, *J* = 7.8 Hz, 4H), 7.73 (t, *J* = 7.8 Hz, 2H); ^1^H NMR (400 MHz, DMSO-*d*_6_): δ 8.33 (d, *J* = 7.7 Hz, 4H), 7.74 (t, *J* = 7.7 Hz, 2H); ^13^C­{^1^H} NMR (101 MHz, DMSO-*d*_6_): δ 177.2, 134.3, 133.8, 131.6, 130.3; HRMS (ESI/Q-TOF) calcd for C_16_H_8_O_4_Na^+^ (M + H_2_ + Na)^+^ (partially reduced form), 287.0315; found, 287.0319.

### Pyrene-4,5,9,10-tetraone from Pyrene-4,5-dione Using RuO_2_·*n*H_2_O/NaIO_4_

Pyrene-4,5-dione (7.15 g, 0.50 mol), RuO_2_·*n*H_2_O (0.72 g, 0.75 mol), NaIO_4_ (35.80 g, 0.17 mol), CH_3_CN (200 mL), and H_2_O (100 mL) were combined in a 1 L round-bottom flask fitted with an efficient stirring magnet. The resulting greenish-brown slurry was stirred vigorously at room temperature for 2 h. The mixture was diluted with 200 mL of H_2_O and the resulting precipitate was collected on a suction funnel. The filter cake was washed rigorously with 3 × 100 mL of H_2_O and dried in the oven at 120 °C to afford the tetraone product (5.07 g, 62%). The greenish tint of the product apparently stems from trace residues of unidentified Ru compounds. The purity of the product was confirmed by TLC (10:1 CH_2_Cl_2_:EtOAc) and NMR analysis, rendering the material suitable for most applications without additional purification.

### Pyrene-4,5,9,10-tetraone from Pyrene-4,5-dione Using H_5_IO_6_

A 250 mL round-bottom flask equipped with a highly efficient stirring magnet and a reflux condenser was charged with pyrene-4,5-dione **1** (5.0 g, 21.6 mmol), H_5_IO_6_ (14.8 g, 64.8 mmol), and glacial acetic acid (200 mL). The resulting brown slurry was stirred at mild reflux (the oil bath surrounding the reaction flask was kept at 118 °C) for 12 h. NMR and TLC (10:1 CH_2_Cl_2_:EtOAc) analysis showed complete conversion of **1** to **2** after this period. Upon cooling to room temperature, the reaction mixture was diluted with excess water, and the solid was collected by filtration and dried in the oven at 120 °C to afford 3.25 g (57%) of the tetraone.

## Supplementary Material



## Data Availability

The data underlying this study are available in the published article and its Supporting Information.

## Notes

General remarks, ^1^H and ^13^C­{^1^H} NMR spectra, high-resolution mass spectra, and FTIR and Raman spectra (PDF)

## References

[1] Vollmann H., Becker H., Corell M., Streeck H. (1937). Beiträge zur
Kenntnis des Pyrens und seiner Derivate. Justus
Liebigs Ann. Chem..

[2] Figueira-Duarte T. M., Müllen K. (2011). Pyrene-Based
Materials for Organic Electronics. Chem. Rev..

[3] Sun H., Wu X., Yao B., Li G., Qi N., Shi L. (2024). Design and Synthesis of Ladder-type Covalent Organic
Frameworks. Arabian J. Chem..

[4] Albold U., Hoyer C., Neuman N. I., Sobottka S., Hazari A. S., Lahiri G. K., Sarkar B. (2019). Isolable Cu­(I)
Complexes of Extremely
Electron-Poor, Completely Unreduced o-Quinone and ″Di- o-Quinone″
Ligands Stabilized through π-π Interactions in the Secondary
Coordination Sphere. Inorg. Chem..

[5] Nokami T., Matsuo T., Inatomi Y., Hojo N., Tsukagoshi T., Yoshizawa H., Shimizu A., Kuramoto H., Komae K., Tsuyama H., Yoshida J. (2012). Polymer-Bound Pyrene-4,5,9,10-tetraone
for Fast-Charge and -Discharge Lithium-Ion Batteries with High Capacity. J. Am. Chem. Soc..

[6] Casas-Solvas J. M., Howgego J. D., Davis A. P. (2014). Synthesis
of Substituted Pyrenes
by Indirect Methods. Org. Biomol. Chem..

[7] Merz J., Dietz M., Vonhausen Y., Wöber F., Friedrich A., Sieh D., Krummenacher I., Braunschweig H., Moos M., Holzapfel M., Lambert C., Marder T. B. (2020). Synthesis, Photophysical and Electronic
Properties of New Red-to-NIR Emitting Donor–Acceptor Pyrene
Derivatives. Chem.—Eur. J..

[8] Oberender F. G., Dixon J. A. (1959). Osmium and Ruthenium Tetroxide-Catalyzed Oxidations
of Pyrene. J. Org. Chem..

[9] Hu J., Zhang D., Harris F. W. (2005). Ruthenium­(III)
Chloride Catalyzed
Oxidation of Pyrene and 2,7-Disubstitued Pyrenes: An Efficient, One-Step
Synthesis of Pyrene-4,5-diones and Pyrene-4,5,9,10-tetraones. J. Org. Chem..

[10] Walsh J. C., Williams K.-L. M., Lungerich D., Bodwell G. J. (2016). Synthesis of Pyrene-4,5-dione
on a 15 g Scale. Eur. J. Org Chem..

[11] Mo, Y. Method for Synthesizing Pyrene-4,5,9,10-tetralone. CN 102617317 A, 2012.

[12] Piccialli V. (2014). Ruthenium
Tetroxide and Perruthenate Chemistry. Recent Advances and Related
Transformations Mediated by Other Transition Metal Oxo-species. Molecules.

[13] Carlsen P. H. J., Katsuki T., Martin V. S., Sharpless K. B. J. A. (1981). Greatly
Improved Procedure for Ruthenium Tetroxide Catalyzed Oxidations of
Organic Compounds. Org. Chem..

[14] El-Assaad T. H., Parida K. N., Cesario M. F., McGrath D. V. (2020). Sterically Driven
Metal-free Oxidation of 2,7-Di-*tert*-butylpyrene. Green Chem..

[15] El-Assaad T. H., McGrath D. V. (2024). Nonsymmetric Pyrene-Fused
Pyrazaacenes via Green Oxidation
of 2,7-Di-tert-butylpyrene. J. Org. Chem..

