# Supplementary material for: Syntheses of Pyrene-4,5-dione and Pyrene-4,5,9,10-tetraone
Source: J Org Chem. 2025 Oct 4;90(41):14835–8. doi: 10.1021/acs.joc.5c01542 (PMC12538577; doi:10.1021/acs.joc.5c01542)

Supporting Information

Syntheses of Pyrene-4,5-dione and Pyrene-4,5,9,10-tetraone

***Omolola Balogun,*[1] *Besan Khader,*[1,2] *Tetyana Ignatova*[2] *and Aleksandrs Prokofjevs*[1*]**

[1]Department of Chemistry, North Carolina A&T State University, Greensboro, North Carolina 27411
[2]Nanoscience Department, The Joint School of Nanoscience & Nanoengineering, University of North Carolina, Greensboro, North Carolina 27401

aprokofjevs@ncat.edu

S2. General Remarks

S3. ^{1}H NMR Spectrum of Pyrene-4,5-dione (**1**) in $CDCl_3$

S5. ^{1}H NMR Spectrum of Pyrene-4,5-dione (**1**) in d_6-DMSO

S7. ^{13}C NMR Spectrum of Pyrene-4,5-dione (**1**) in $CDCl_3$

S9. ^{13}C NMR Spectrum of Pyrene-4,5-dione (**1**) in d_6-DMSO

S11. ^{1}H NMR Spectrum of Pyrene-4,5,9,10-tetraone (**2**) in $CDCl_3$

S13. ^{1}H NMR Spectrum of Pyrene-4,5,9,10-tetraone (**2**) in d_6-DMSO

S15. ^{13}C NMR Spectrum of Pyrene-4,5,9,10-tetraone (**2**) in d_6-DMSO

S17. ESI/QTOF HRMS Characterization of **1** and **2**

S18. FTIR Spectra of **1** and **2**

S19. Raman Spectra of **1** and **2**

General Remarks

All reactions were performed using standard laboratory techniques. No effort was made to protect the reaction mixtures from exposure to air or moisture. Commercial HPLC grade methylene chloride and acetonitrile solvents were used without further purification. Pyrene, potassium carbonate, sodium sulfite, acetic acid, benzonitrile, sodium periodate, H_5IO_6, CrO_3 (all from Thermo Scientific) and $RuO_2 \cdot nH_2O$ (Alfa Aesar) were used as received. Commercial grade potassium pesulfate (Thermo Scientific) was ground using a mortar and pestle in case the crystals were too large (see Experimental Section).

Nuclear magnetic resonance spectra were recorded using Bruker Ascend 400 MHz spectrometer in $CDCl_3$ or d_6-DMSO solution at 298K and referenced to the signal of the internal Me_4Si. Fourier transform infrared spectra were recorded on solid samples using Shimadzu IRTracer-100 spectrometer equipped with an attenuated total reflectance attachment. Advanced ATR correction and multipoint baseline correction were applied to the IR spectra. Raman spectra were acquired using HORIBA XploRa Raman Confocal system. The laser excitation wavelength was 532 nm, and a 1200 mm^{-1} diffraction grating was used.

^{1}H NMR (400 MHz)
$CDCl_3$

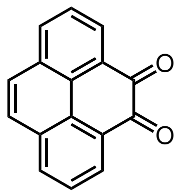

1

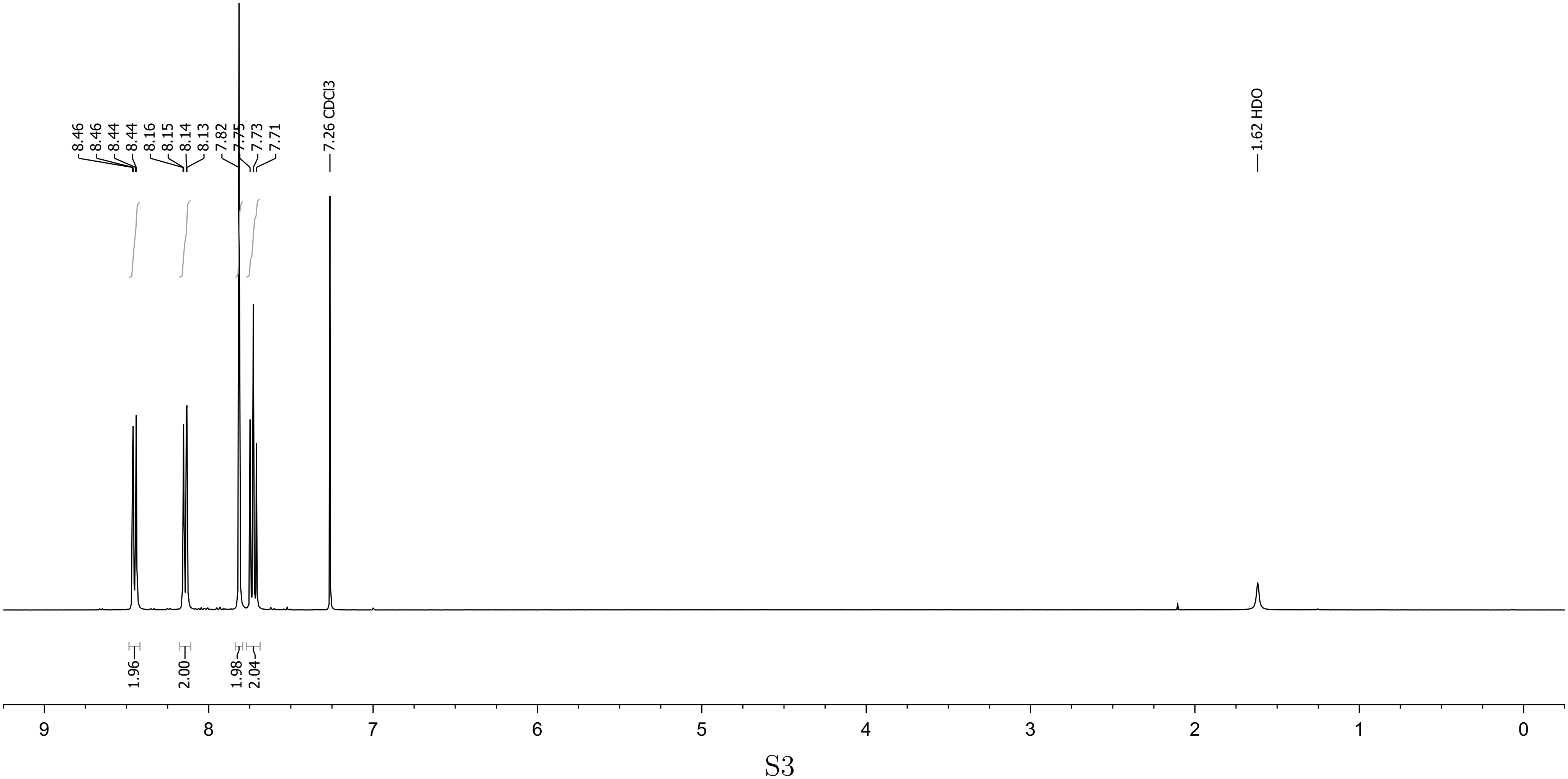

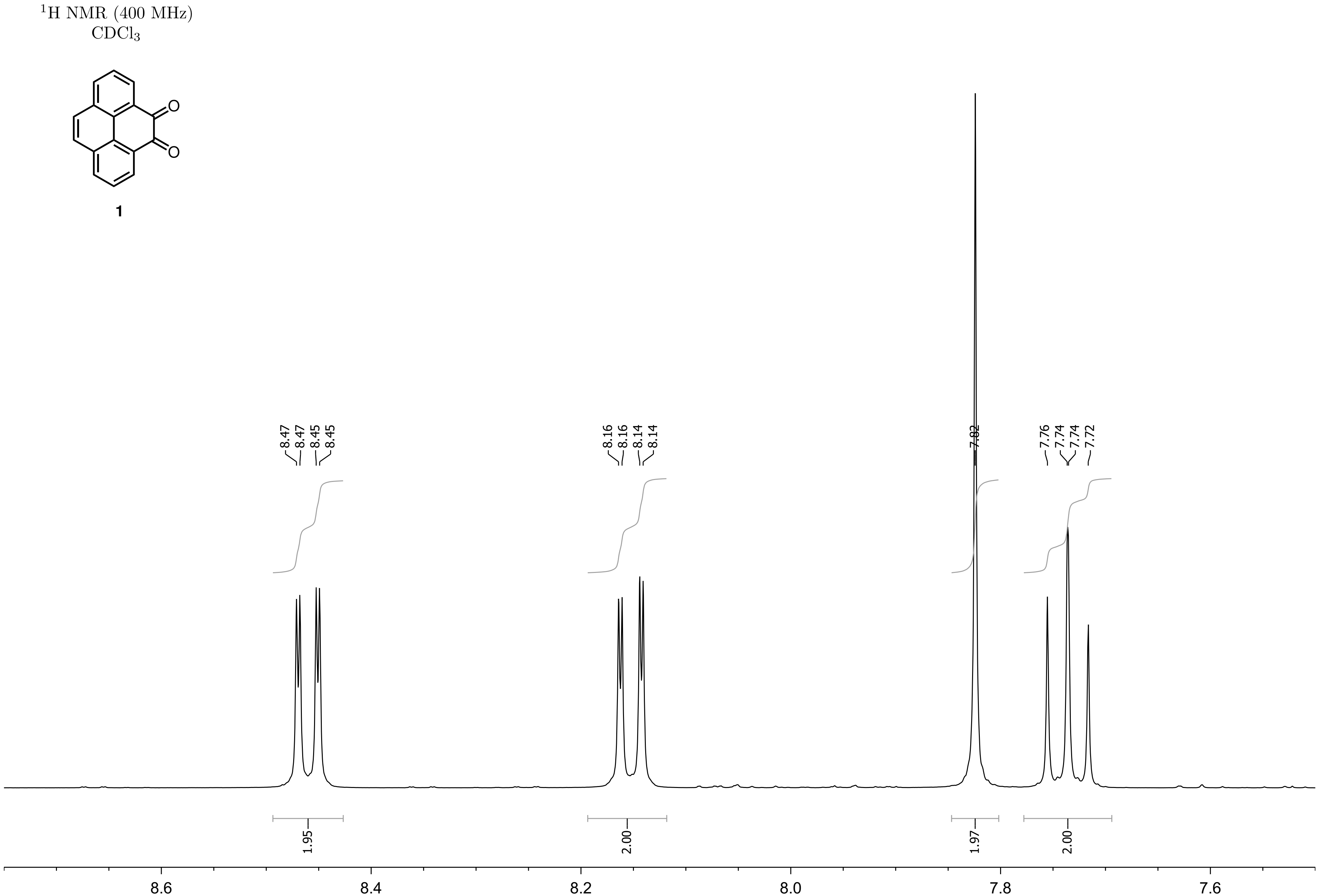

¹H NMR (400 MHz)
CDCl3
1
8.47
8.47
8.45
8.45
8.16
8.16
8.14
8.14
7.82
7.76
7.74
7.74
7.72
1.95
2.00
1.97
2.00
8.6
8.4
8.2
8.0
7.8
7.6

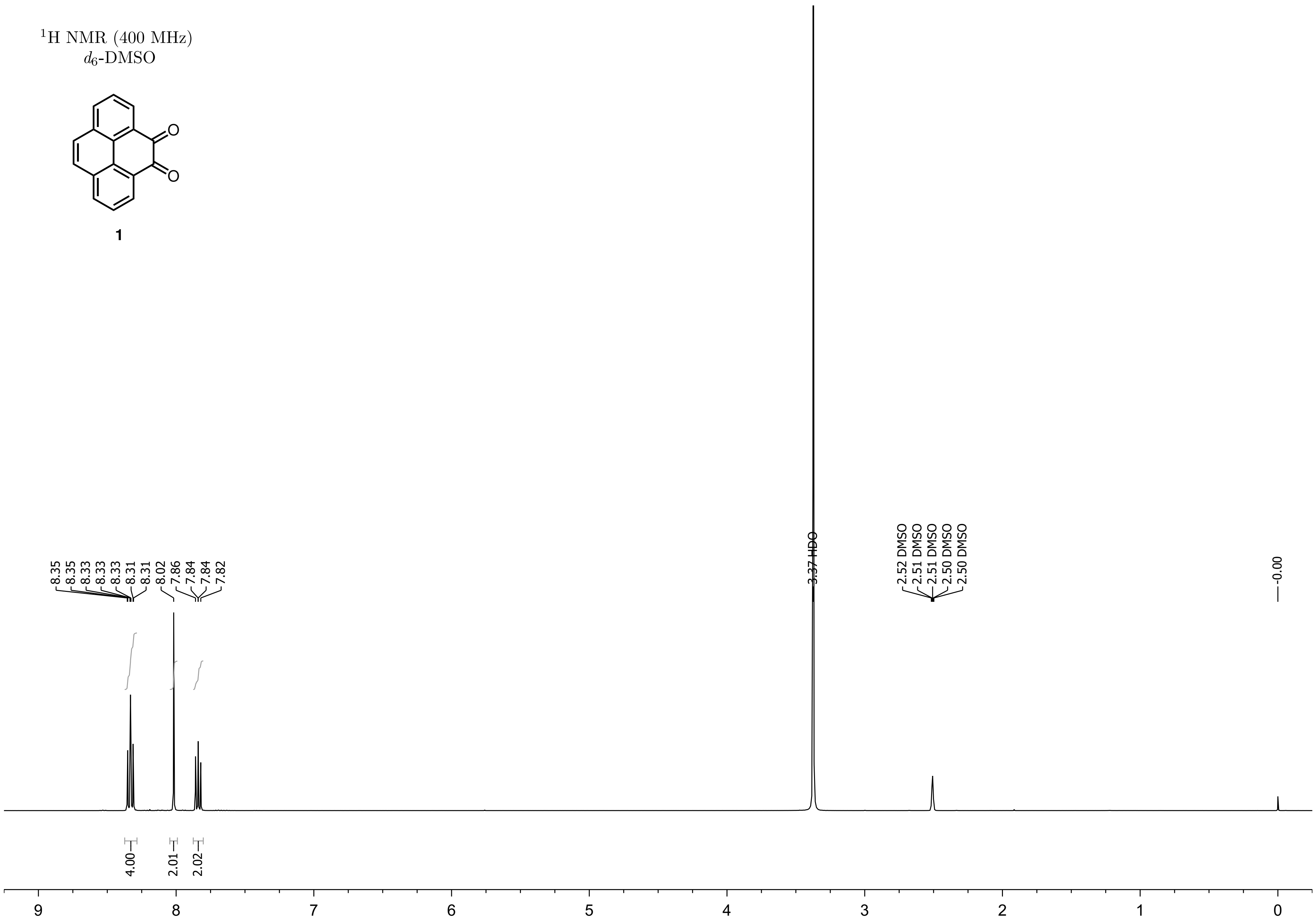
^{1}H NMR (400 MHz)
d_6-DMSO
1
8.35
8.35
8.33
8.33
8.33
8.31
8.31
8.02
7.86
7.84
7.84
7.82
3.37 HDO
2.52 DMSO
2.51 DMSO
2.51 DMSO
2.50 DMSO
2.50 DMSO
-0.00
4.00
2.01
2.02
9
8
7
6
5
4
3
2
1
0

^{1}H NMR (400 MHz)
d_6-DMSO

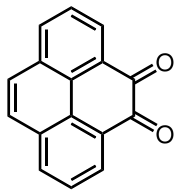

1

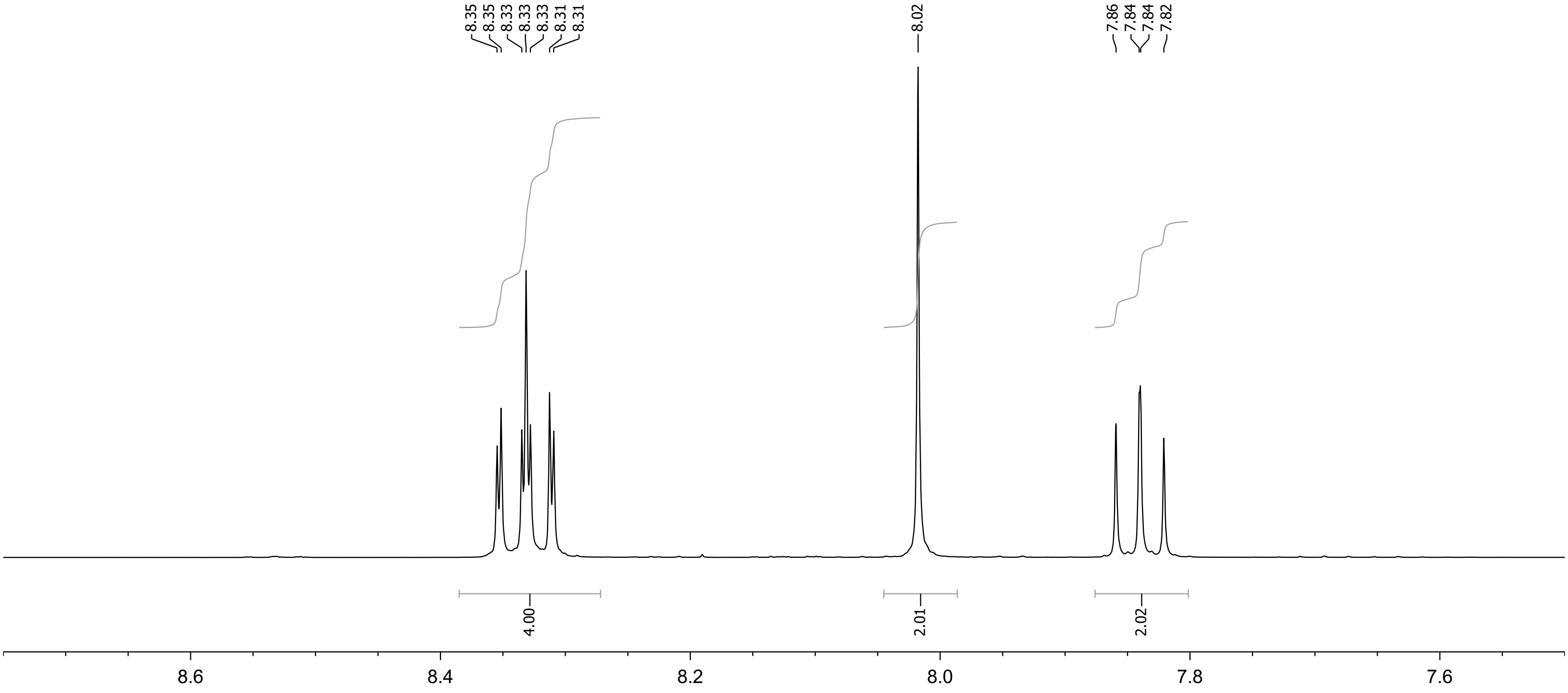

$^{13}C\{^{1}H\}$ NMR (101 MHz)
$CDCl_3$

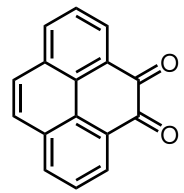

1

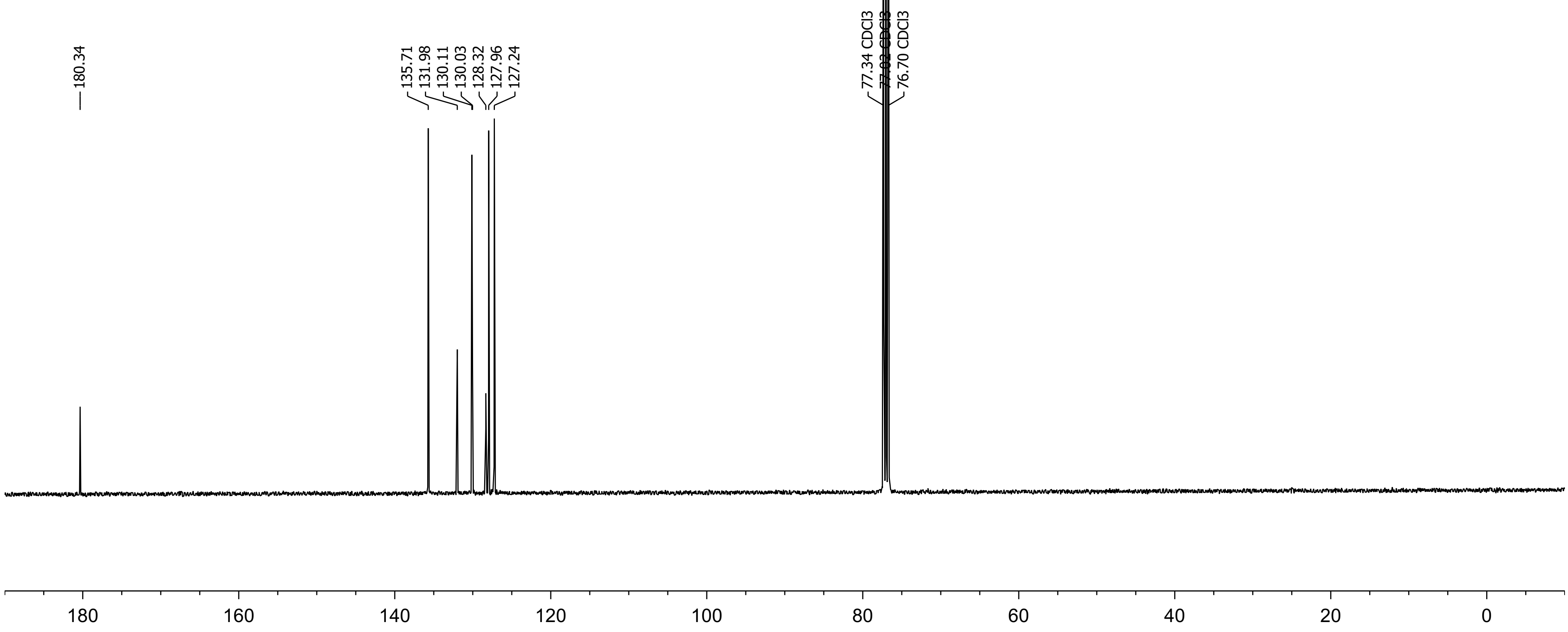

$^{13}C\{^1H\}$ NMR (101 MHz)
$CDCl_3$

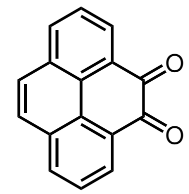

1

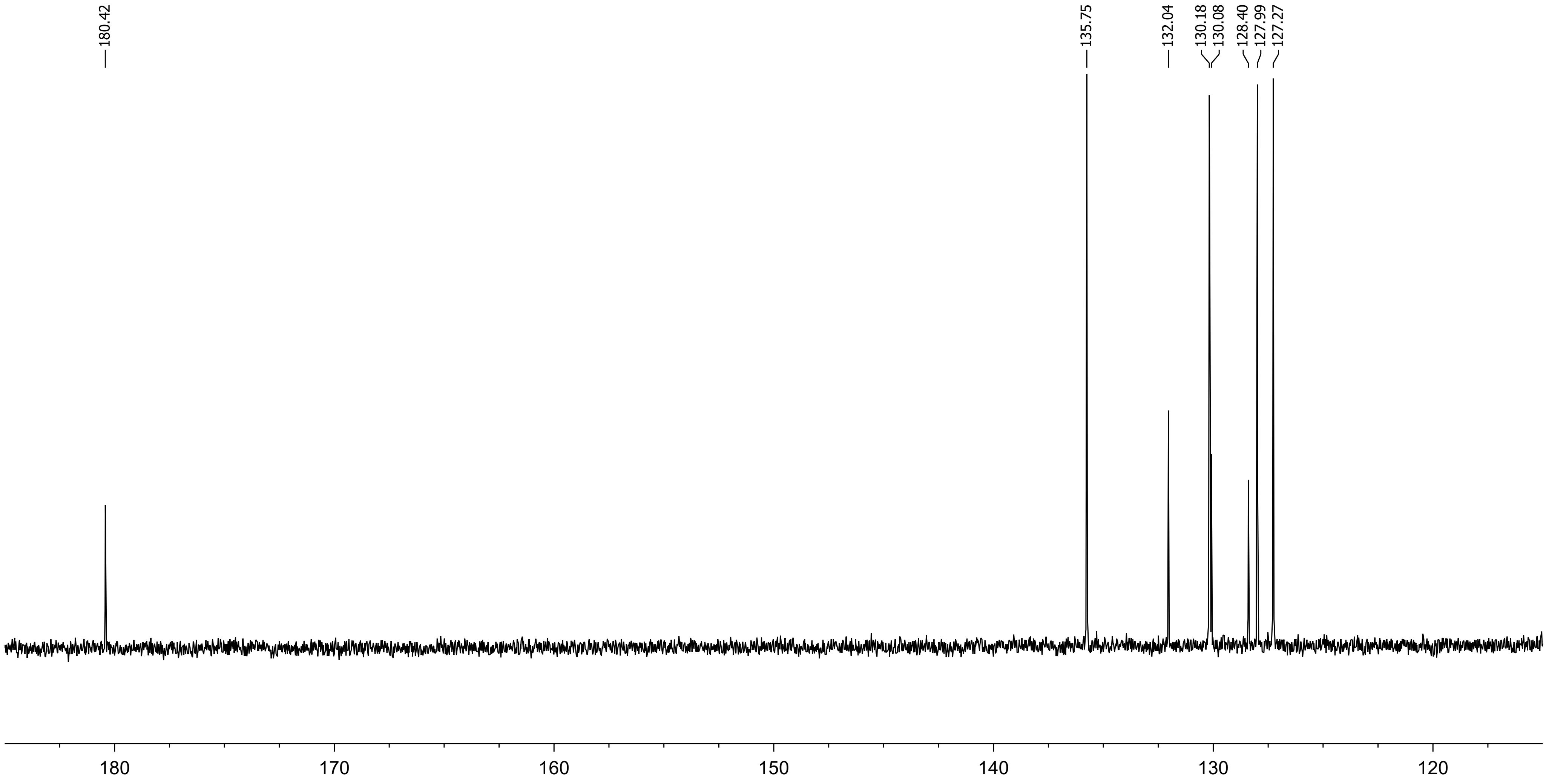

$^{13}C\{^{1}H\}$ NMR (101 MHz)
d_6-DMSO

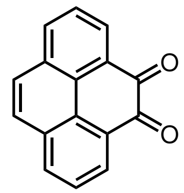

1

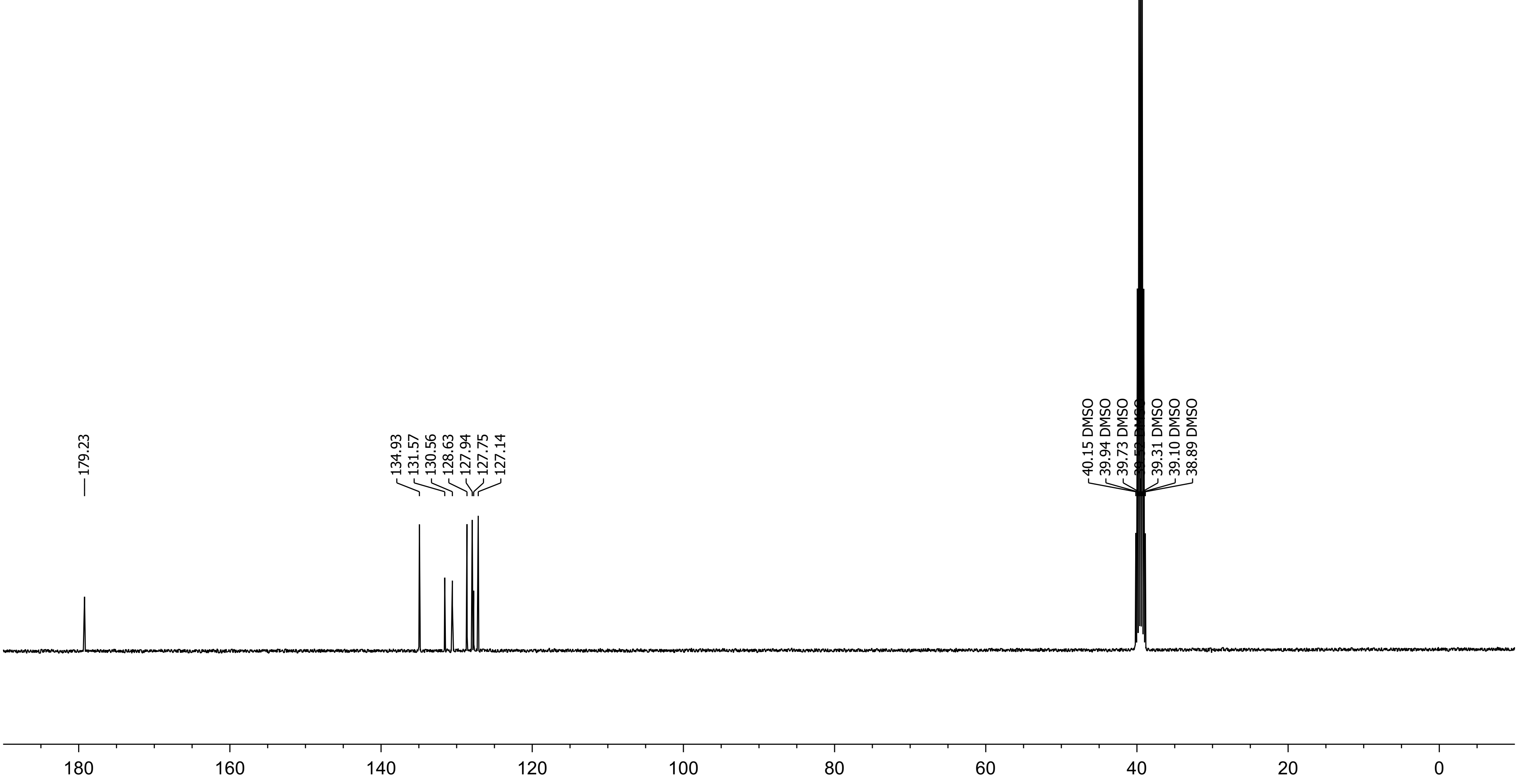

$^{13}C\{^{1}H\}$ NMR (101 MHz)
d_6-DMSO

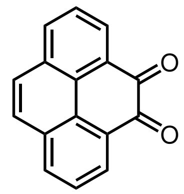

1

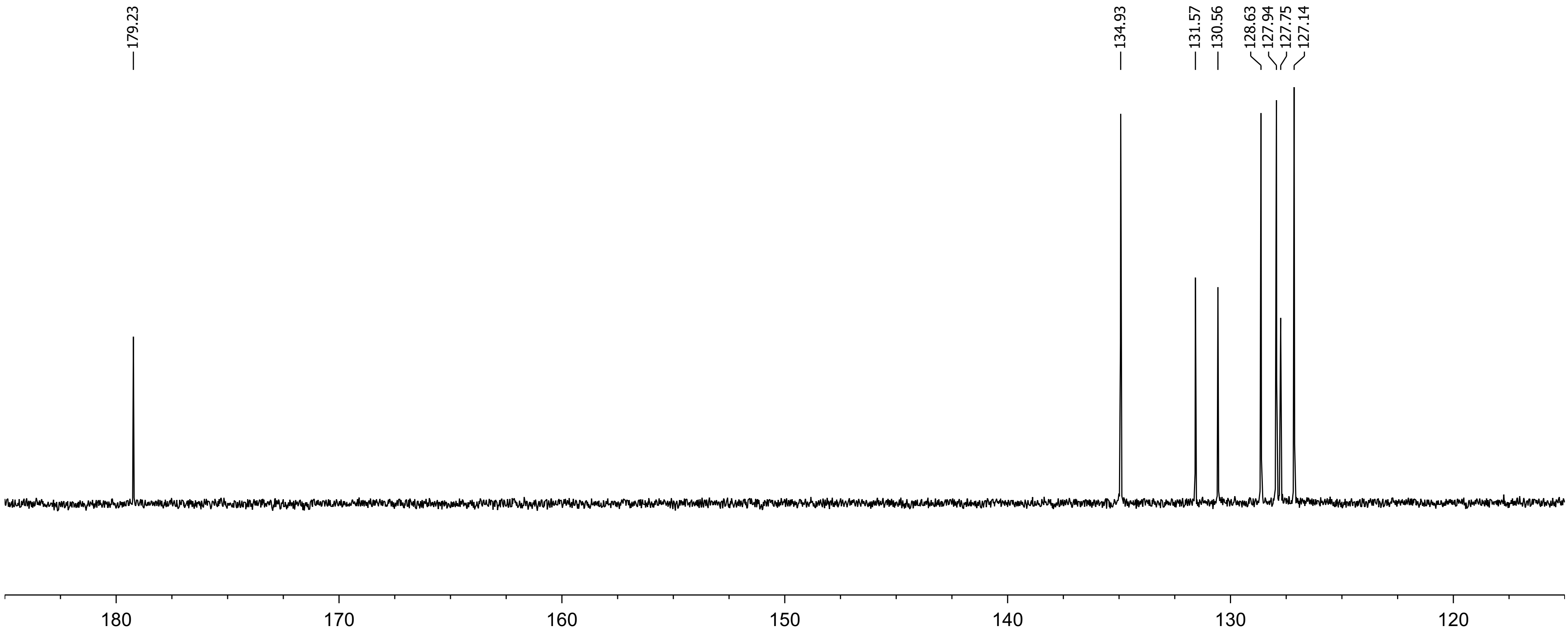

^{1}H NMR (400 MHz)
$CDCl_3$

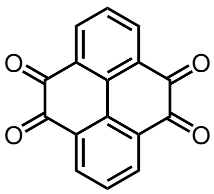

2

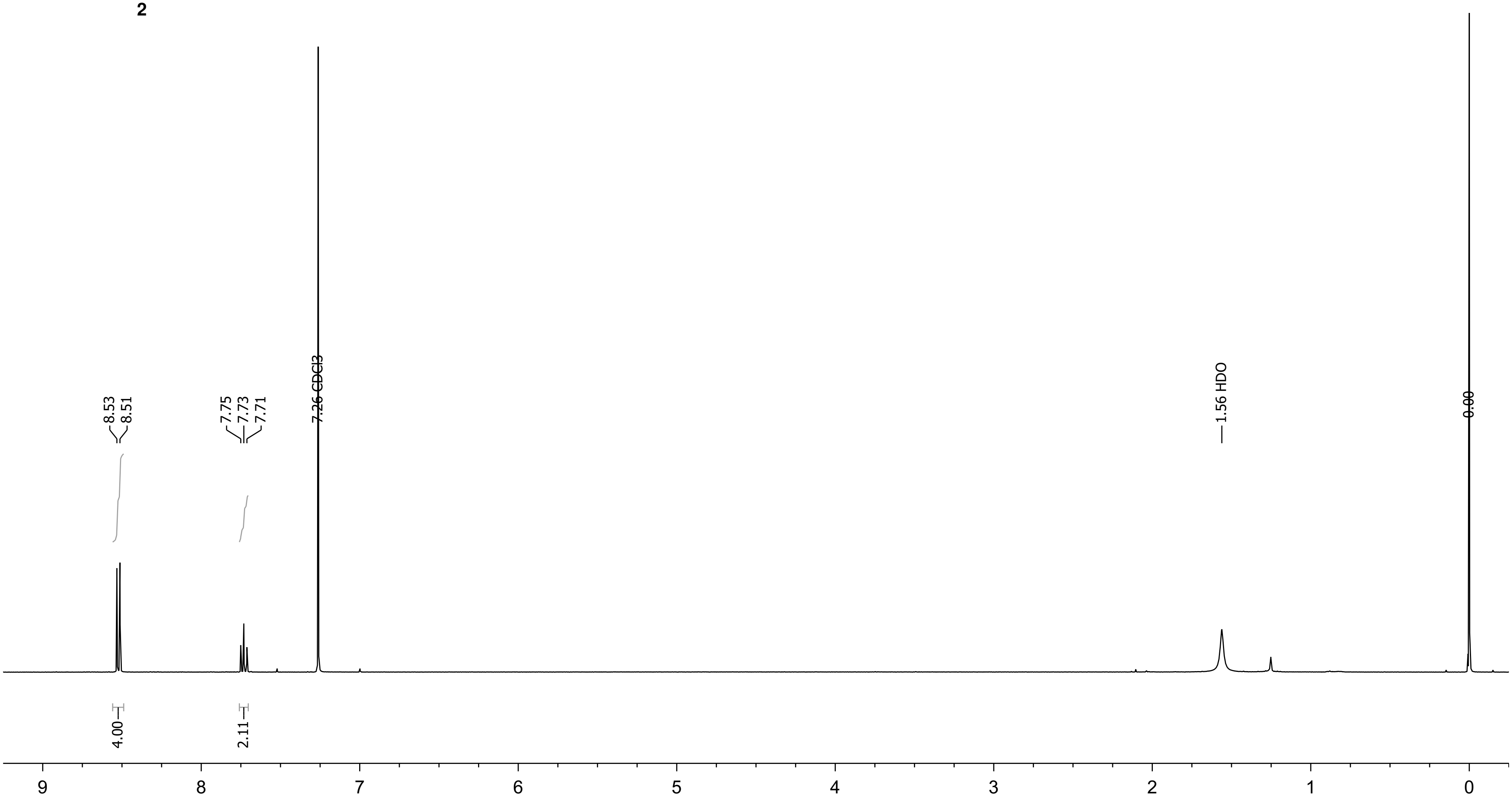

^{1}H NMR (400 MHz)
$CDCl_3$

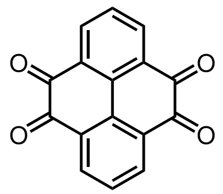

2

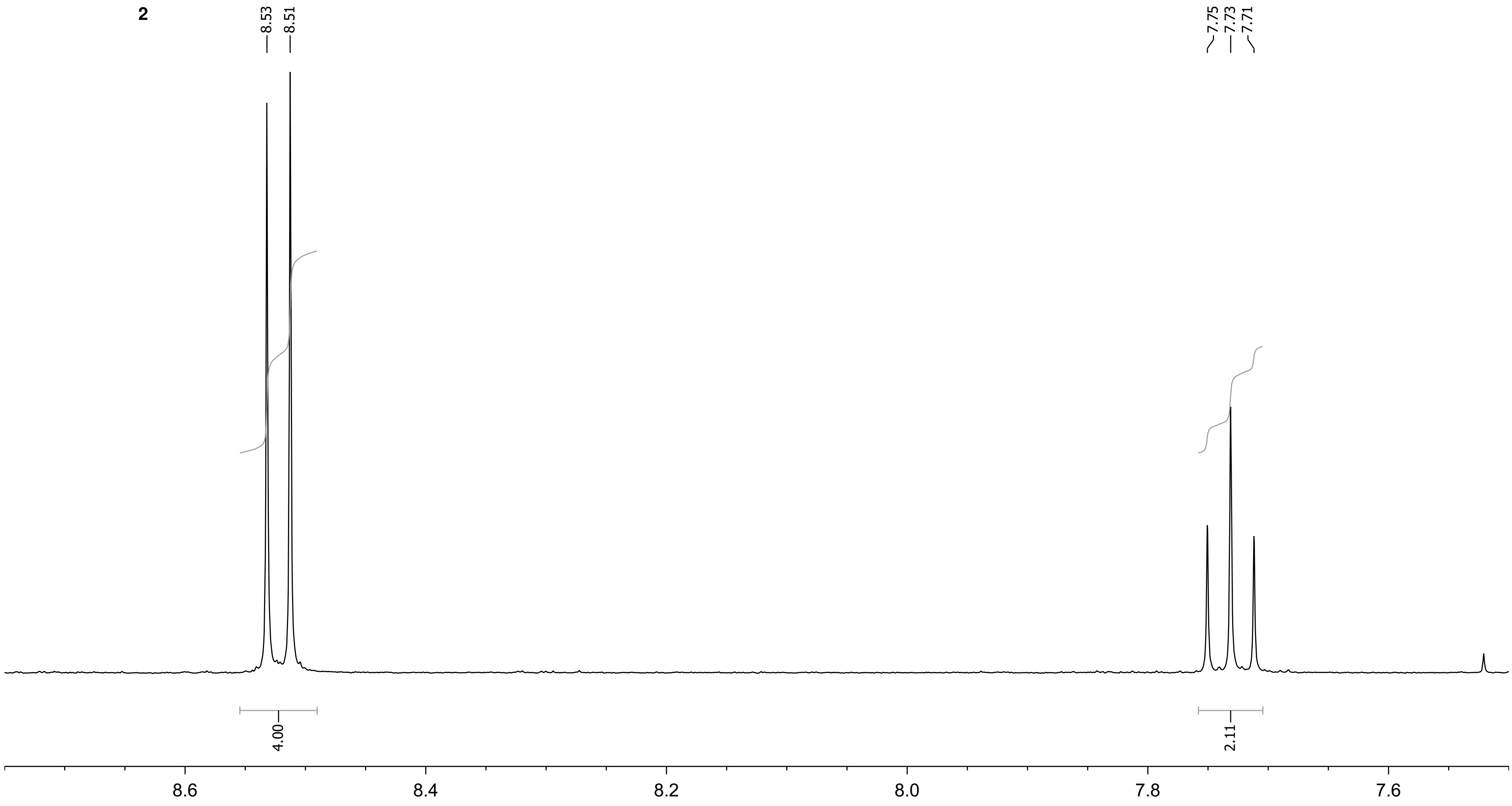

^{1}H NMR (400 MHz)
d_6-DMSO

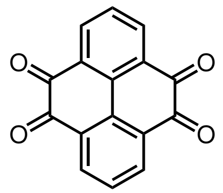

2

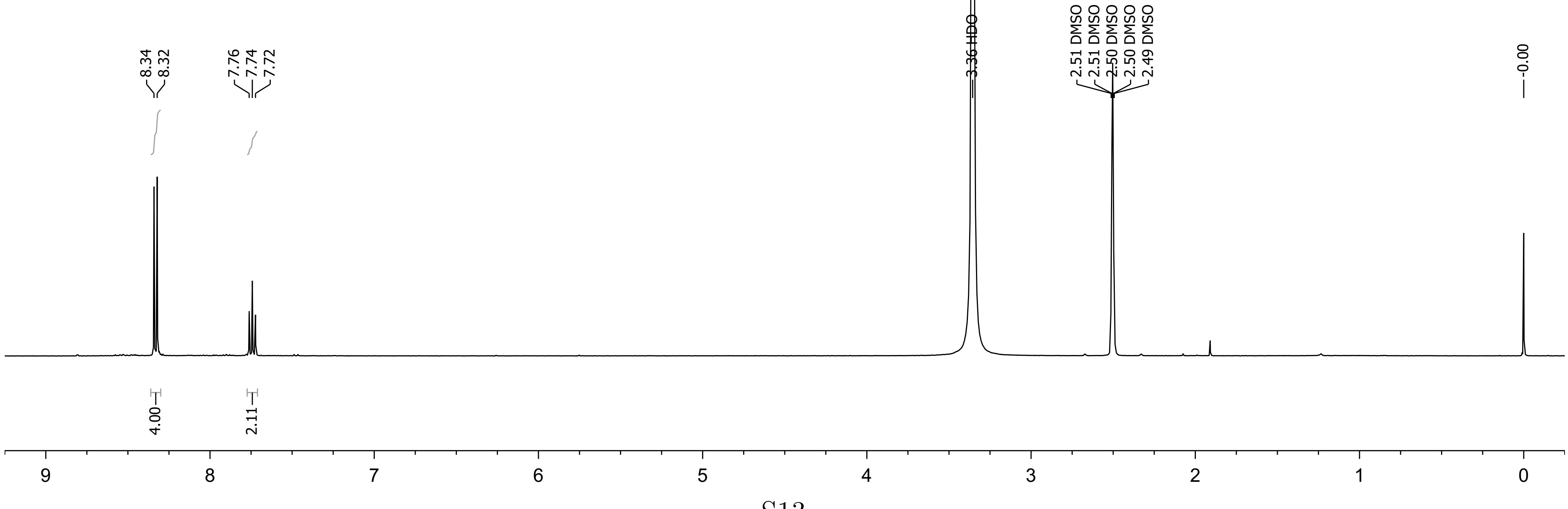

^{1}H NMR (400 MHz)
d_6-DMSO

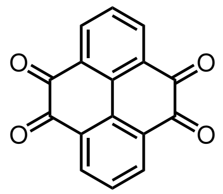

2

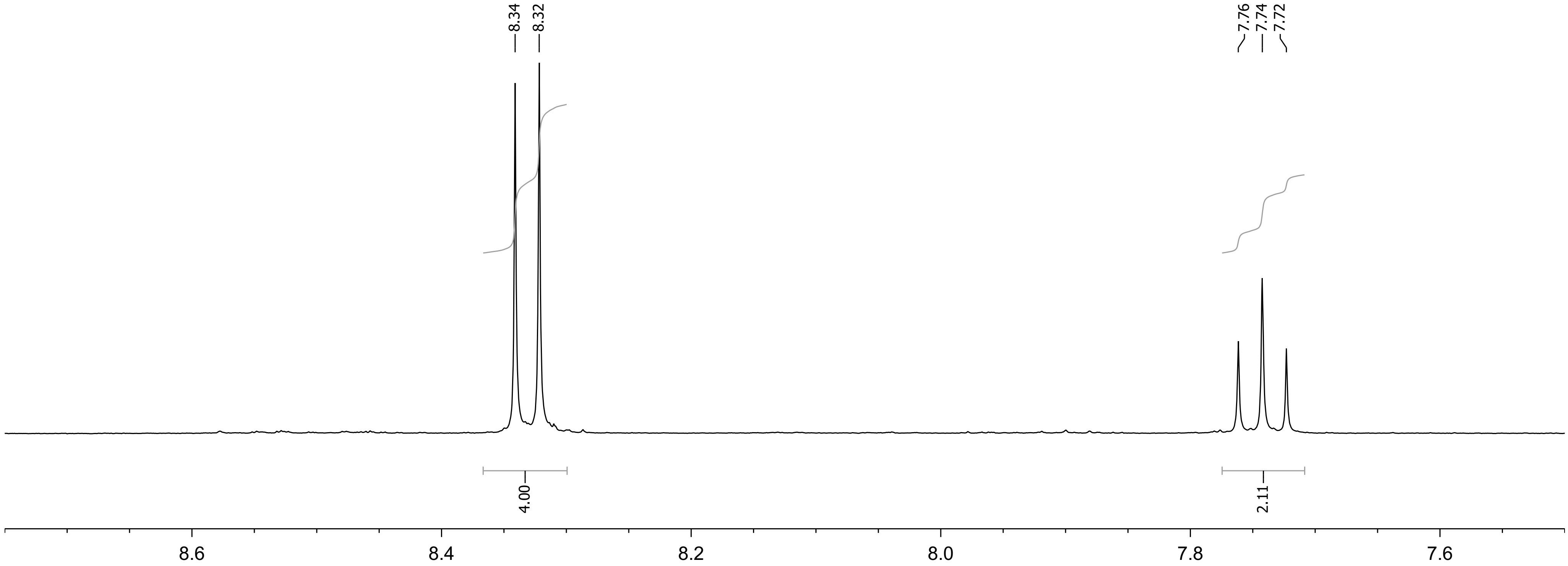

$^{13}C\{^{1}H\}$ NMR (101 MHz)
d_6-DMSO

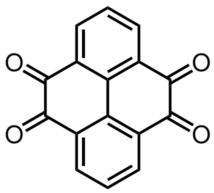

2

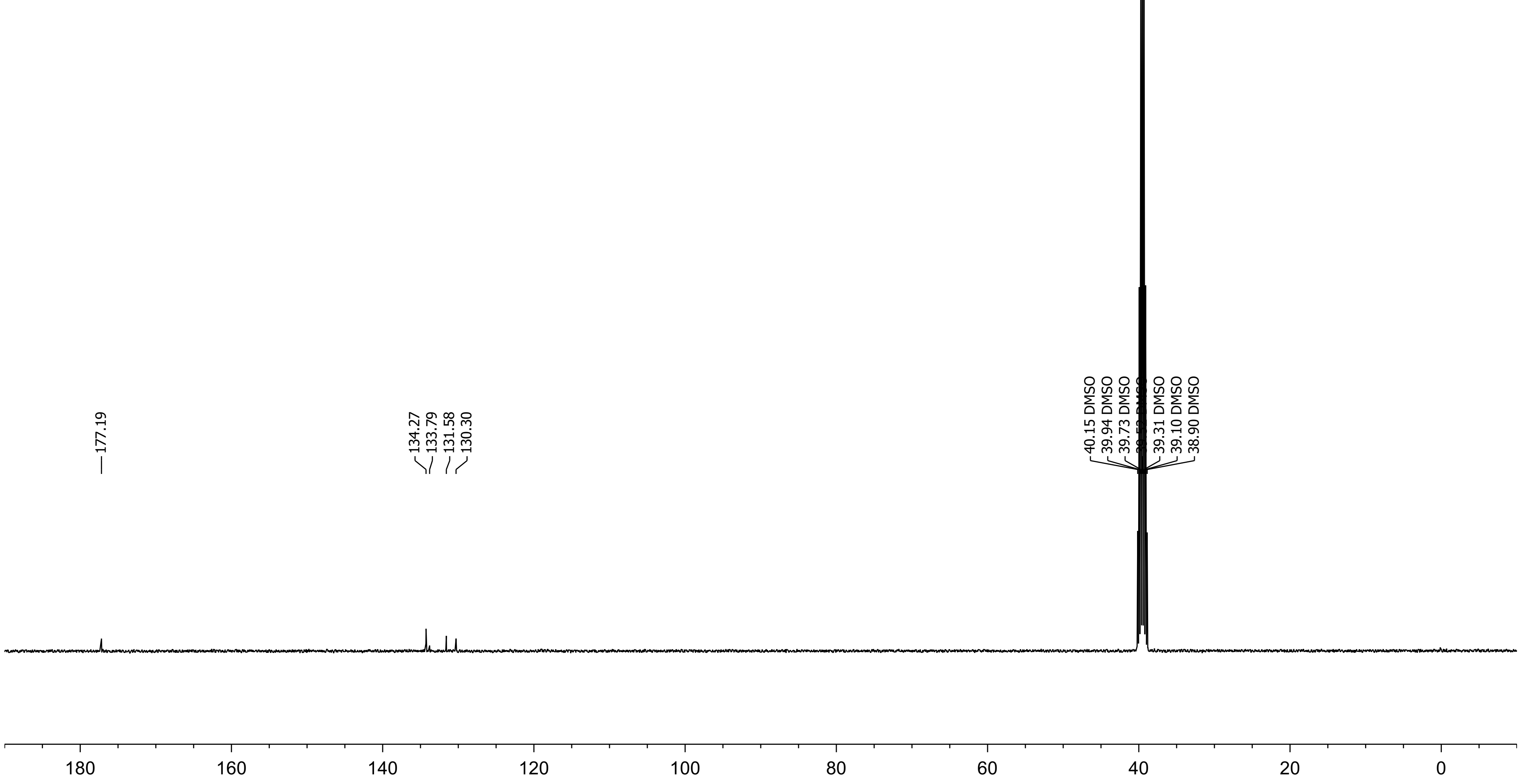

$^{13}C\{^{1}H\}$ NMR (101 MHz)
d_6-DMSO

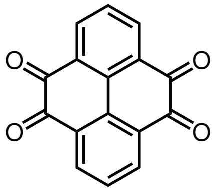

2

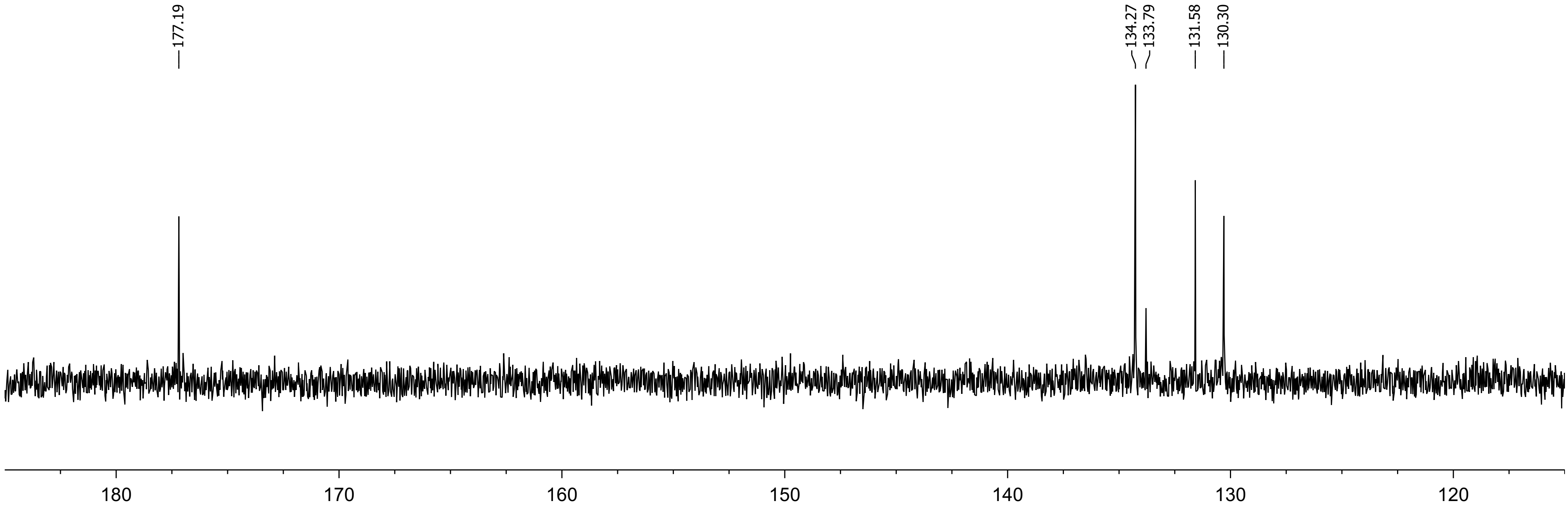

ESI/QTOF HRMS Characterization of **1** and **2**

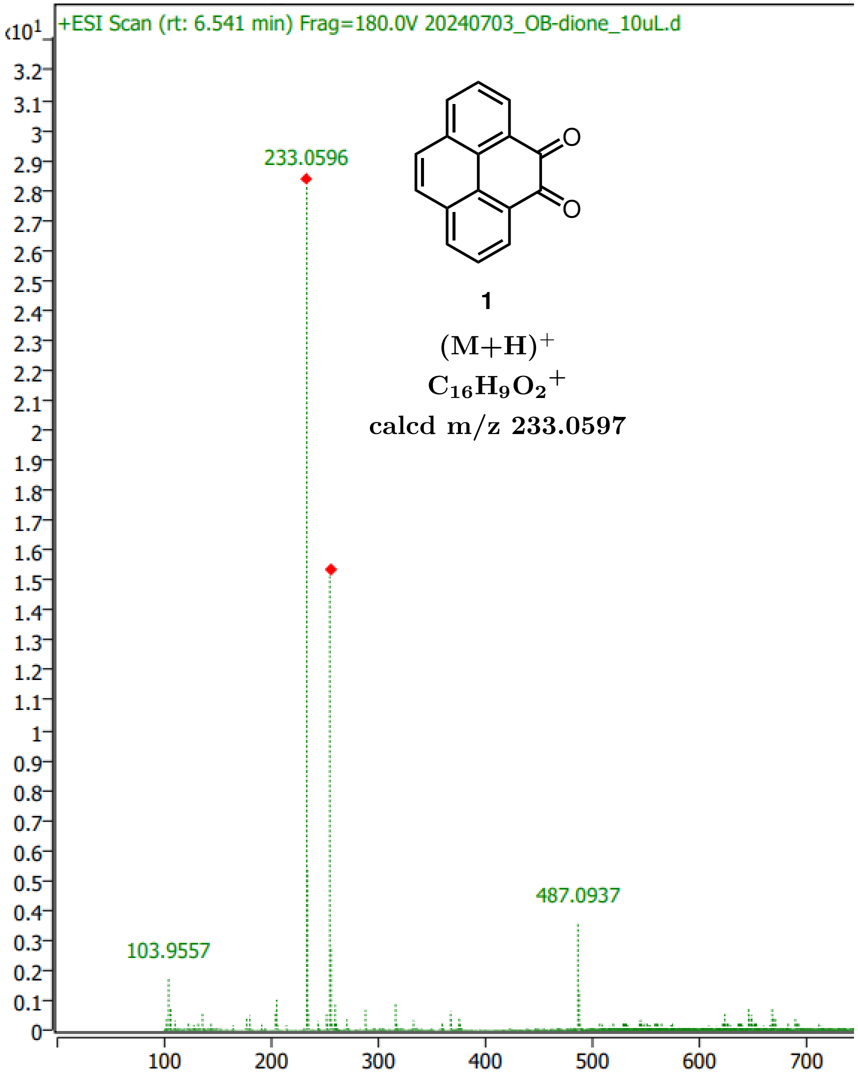

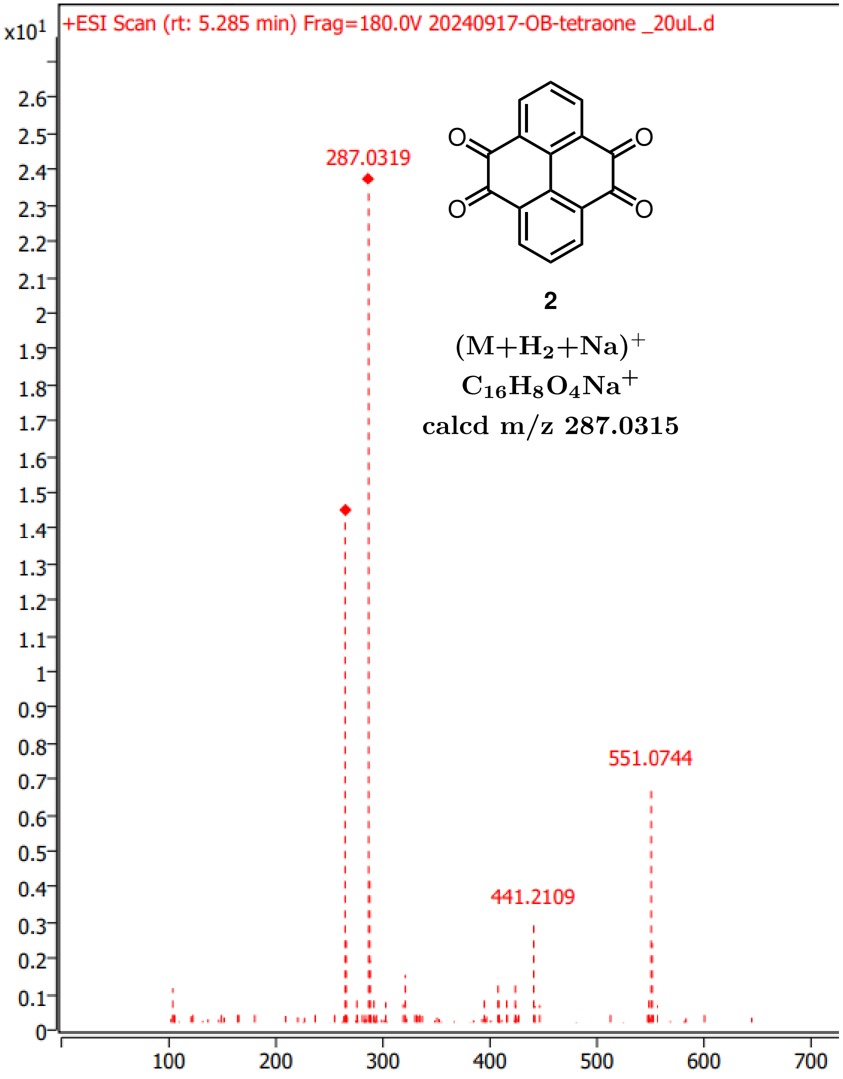

ATR-FTIR Spectra of **1** and **2**

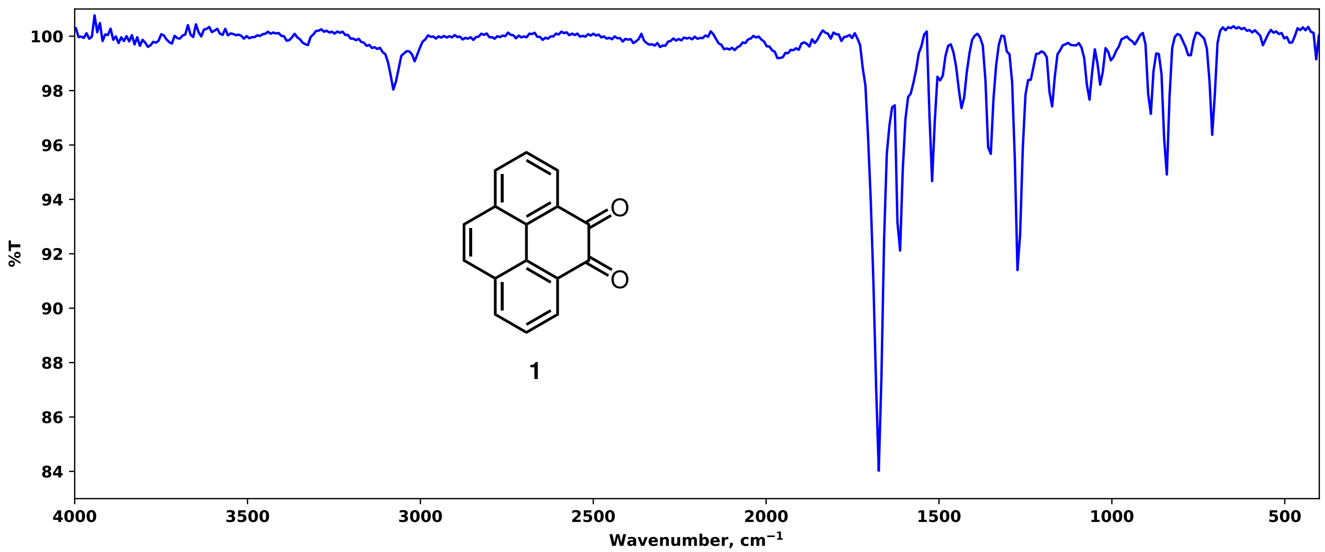

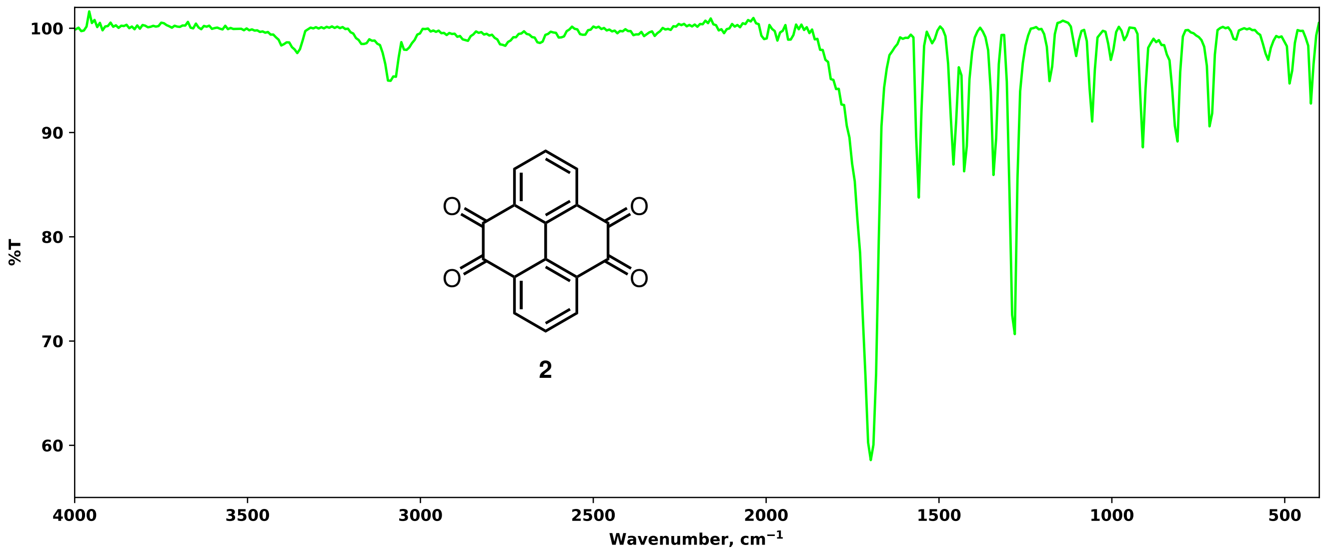

Raman Spectra of **1** and **2**

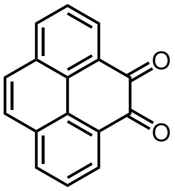

1

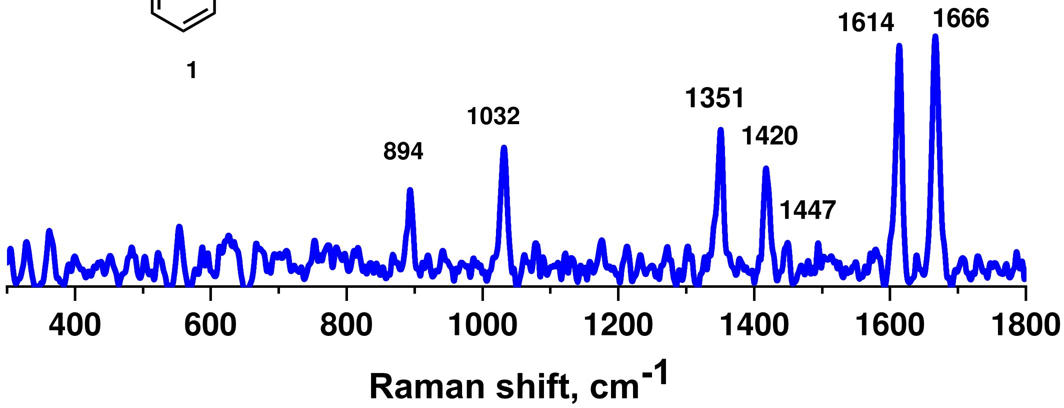

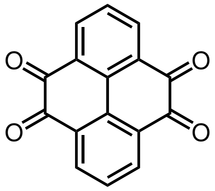

2

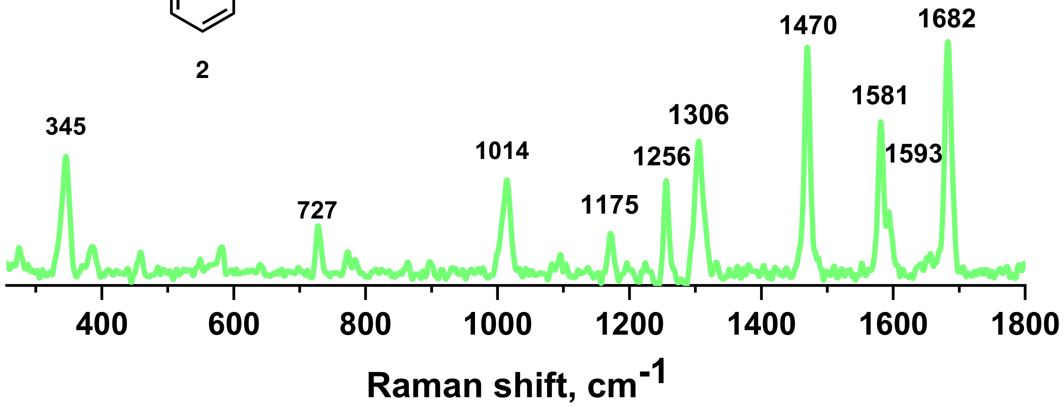

Supplement: Supplementary file 1 [file jo5c01542_si_001.pdf]
